# The clinical practice of oncology nursing in Brazil: realities and challenges in the training of specialist nurses

**DOI:** 10.3332/ecancer.2014.ed46

**Published:** 2014-11-03

**Authors:** Maria Gaby Rivero de Gutiérrez, Adriana Maria Duarte, Carla Gonçalves Dias

**Affiliations:** 1 Paulista School of Nursing, Federal University of São Paulo (Escola Paulista de Enfermagem, Universidade Federal de São Paulo), São Paulo, SP, Brazil; 2 Graduate Nursing Program of the Paulista School of Nursing of the Federal University of São Paulo (Escola Paulista de Enfermagem, Universidade Federal de São Paulo), EPE–UNIFESP, São Paulo, SP, Brazil; 3 Manager of Education and Research, Institute of Pediatric Oncology (Instituto de Oncologia Pediátrica–IOP/GRAACC-UNIFESP), São Paulo, SP, Brazil

Health indicators show cancer to be a public health problem around the world, including Brazil. In our country, the estimate for the year 2014 predicts the occurrence of approximately 576,000 new cases of cancer, representing the second leading cause of death among men and women. The highest rates recorded in Brazil are in the South and Southeastern regions (116,330 and 299,730, respectively), while the lowest are in the North and Northeastern regions (20,020 and 99,060, respectively). The Midwest region shows an intermediate pattern [1].

Under Brazilian law, ‘health is a right of every citizen, and the duty of the State’ [2]. In order to guarantee this right, health care for the population, including cancer care, is provided through services linked to the Unified Health System (‘SUS’), registered with the Ministry of Health as High Complexity Oncology Centers (‘CACON’). These High Complexity Oncology Centers are government or philanthropic hospitals that have the human and technological resources needed to provide full care to cancer patients. In this context, to provide care to individuals with the suitable structure, with the best treatment standards, and assuring the best chances of cure, can only be achieved with qualified and specialized professionals [3].

With regard to nursing, the increased demand for oncology services consequently encouraged the development of a specialty in the area. This in turn led to the emergence of oncology nursing organizations, the need for undergraduate courses to include oncology in their curricula, and to offer specialization, refresher and extension courses, among others. From this perspective, it is essential to reflect on the nursing practice in terms of its requirement for broad knowledge, from a scientific, technological and humanistic perspective, with regard to the different care demands that cancer patients and their families experience throughout the illness process.

To become a specialist in Brazil at least 360 hours of training are required, which include theoretical and practical knowledge of the specialty. As for training the specialist in the type of residence, the workload is much higher (5,760–4,608 hours in practical and 1,152 hours in theoretical knowledge), and is characterized by in-service training, with an emphasis on clinical practice. In general, the contents taught cover: epidemiologic and bioethical issues in oncology; conceptual basis and diagnostic tools applied in oncology; treatment modalities; oncological pathologies; oncological emergencies; palliative care and symptom control; and management in oncology.

Training and specialization in nursing continues to be inconsistent in relation to the specific content related to cancer by the time of graduation, and there is a gap between the technical-and-scientific preparation and the practice of specialized care. The importance of extending the qualification of health professionals is further strengthened given the figures, which show that out of the 420 accredited nursing schools in the most populous and developed region of the country - southeastern Brazil - only 31 of them offer specialized oncology nursing courses [4].

With regard to specialization courses specifically geared towards the pediatric population affected by cancer, the scenario is even more worrying since there are practically no courses in this area. Thus, the acquisition of specific skills to care for this population is achieved by a large contingent of nurses, in the course of their health care practice.

Regulatory and reference instances in Brazilian Oncology - the Brazilian Society of Oncology Nursing (‘SBEO’) and the *José Alencar Gomes da Silva* National Cancer Institute (‘INCA’), have also made efforts to train and qualify human resources devoted to cancer care, though it is still very short of what is needed to meet the demand from oncology services and patients. Data provided by the Brazilian Society of Oncology Nursing show that, between 2005 and 2013, only 150 nurses were certified as oncology specialists, i.e. 16 to 17 professionals per year. Likewise, the data on the training of nurses specialized in oncology from the National Cancer Institute (*INCA*), in the residence category, show a lesser number of graduates per year, given that the program has graduated only 103 specialists since its start [5].

Another aspect to consider is that, although oncology nursing has been recognized as a nursing specialty since a little over ten years ago, through Resolution No. 290 of the Federal Nursing Board (‘COFEN’), dated March 24th, 2004 [6], nursing practice in the field of oncology still lacks legal support to ensure the presence of a specialist in the different aspects of oncology practice [7].

In spite of these difficulties, the country does have isolated centers of excellence that value oncology specialist nurse training when hiring their professionals, and which maintain continuing-education programs to enable high-quality nursing services.

One of these experiences is the one developed by the Institute of Pediatric Oncology of the Support Group for Teenagers and Children with Cancer - Federal University of São Paulo (‘IOP/GRAACC-UNIFESP’), by adapting the North American Clinical Nursing Specialist - CNS model to the local reality. For the organization and implementation of this model they adopted the skills proposed by the Association of Pediatric Hematology/Oncology Nurses (‘APHON’), so that nurses could provide qualified aid to patients and their families. Such skills include: direct care; nursing consultation; leadership; collaboration and cooperation with health staff and users; teaching; research; and active participation in ethical and moral decisions [8].

The profile defined by the institution's nursing staff for the clinical nurse specialist role and the title of specialist in Pediatric Oncology, or Oncology Nursing, or Pediatric Nursing, requires passing the training courses in anticancer chemotherapy and totally implantable central venous catheter; Minimum of two years experience in the care of children and teenagers with cancer in outpatient clinical and hospital care; and the strong recommendation that the nurse is participating in a *stricto sensu* graduate [or post-graduate] nursing program [8].

Over the four years of existence of the service organizational model, we have witnessed the undeniable positive influence of the clinical nursing specialist's actions in patient and family responses to care in different situations, as well as the improvement of the care system’s processes dedicated to these clients [8].

For these reasons, the challenges to be faced to ensure the clinical practice of oncology nursing specialists involve aspects related to training as well as policies on the provision of health care that emphasize quality and patient safety. Therefore, it is essential that educational and service institutions rethink their strategies and priorities in order to facilitate the introduction of changes in health work practices, joining forces to create academic and continuing-education processes to meet the reality of the services, and the health care demands of cancer patients and their families.
